# Effects of mind mapping based on standardized patient program in patient education among postgraduate nursing students in clinical setting

**DOI:** 10.1186/s12909-023-04944-4

**Published:** 2023-12-20

**Authors:** Lianhong Wang, Yousha Wang, Xueting Wang, Caixiu Xue

**Affiliations:** Nursing Department of Affiliated Hospital of Zunyi Medical University, ZunYi, Guizhou 563000 China

**Keywords:** Patient education postgraduate nursing education mind mapping standardized patient

## Abstract

**Background:**

Patient education as an important process of postgraduate nursing education, however in previous studies there was limited study focus on the improvement of nursing students’ patient education in clinical practice.This study examined the effects of a mind mapping based on standardized patient program in the patient education knowledge and communication competence of postgraduate nursing students in clinical setting.

**Methods:**

The present quasi-experimental study was performed in 2022 on 74 postgraduate nursing students who had taken clinical practice courses at affiliated hospital of Zunyi Medical University. Students were underwent two weeks of mind mapping based on standardized patient program. The outcome measures were patient education knowledge and communication competence evaluated were by the self-designed questionnaire consisting of 6 questions based on the Likert scale and nurse-patient communication competency rating scale respectively, self-efficacy was evaluated by the general self-efficacy scale, and patients’ satisfaction were measured using a self-designed question. Data collection was conducted before and after intervention. Data analysis was performed using SPSS 19.0 software, and descriptive statistics and inferential statistics were performed.

**Results:**

Significant improvements in patient education knowledge, patient education communication competence, and self-efficacy (all *P* = 0.000)were observed after intervention. Improvements were also seen in measures of patients’ satisfaction; 12/74 (16.22%) patients reported satisfied at baseline but only 53/74 (71.62%) at the end of intervention.

**Conclusions:**

A web-based mind maps integrated with standardized patient program could improve patient education knowledge, communication competence,and self-efficacy of postgraduate nursing students in clinical setting.

## Introduction

Postgraduate education programs were regarded as advanced in higher nursing education, it makes the nursing graduates to equip themselves with more specialized skills and enhance their ability to provide advanced practice services to patients [1]. Advanced practice nurse(APN) is the main direction for postgraduate education program. International Council of Nurses (ICN) address the APN as who has acquired the expert knowledge base, complex decision-making skills and clinical competencies for expanded practice [2]. Direct clinical practice is the central competence for APN [3]. Therefore learning in clinical settings is critical period of postgraduate education programs, which needs to constantly evolve to cultivate highly competent nurse practitioners who can provide safe and good quality patient care [4] .

Patient education as an important process in clinical practice is conducted from the patient’s admission to discharge, and it is performed to improve patient’s knowledge and behavior in ways to achieve better health care outcomes [5]. Abundant research evidences suggest that patient education may improve patient’s satisfaction, empowerment and self-care, quality of life, and also reduce complications and costs related to illness [6–8]. Patient education is a primary responsibility of nurses, especially the professional counseling and coaching consultation is the basic competencies for APN. So it is the paramount need to prepare nursing students to learn the ability of patient education [6]. In the past years, many descriptive studies show that the undergraduate nursing students lack skills necessary for engaging in effective patient education, and need to design instructional strategies that deepen students’ extant knowledge and skills in patient education prior to graduation from nursing programmes [9–12]. Some instructional strategies by drawing on educational and clinical practice guidelines, research and theories was developed to assist nursing students in learning patient education [13–15]. However, these studies have some limitation such as, lacking the evidence for the extent students are integrating theoretical and research underpinnings of patient education into practice, and not addressing the educational efforts [9]. What’s more, in previous studies the study population mainly focus on undergraduate students, while as a postgraduate nursing students who not only need to master the basic knowledge and skill for patients’ health education, but more important to make health education more professional and effectively. Therefore it is important to improve postgraduate nursing students’ competence of integration/understanding/mastery of disease-related knowledge/information, and communication skill with patients.

Mind map as a learning memory tool origins from the full brain actively thinking of associations driven from a central concept [16], and serves as a simple and efficient visual-spatial method/tool of combining drawings with words to build memory links between a topic keyword and image, color, or other link, thereby highlighting the key point and level of the memory contents, allowing learners to effectively store and extract information [17]. Mind map was widely used in nursing education field, and a number of studies demonstrate that it has many advantages, such as facilitating the integration of knowledge and information, and improving students’ problem solving, critical thinking skill, and increases motivation [18–20], and these characteristics help students to provide timely and effective patient education. With the development of technology, mind maps have been used in the computer environment [21, 22]. Compared with the traditional mind map the web-based mind map has many advantages, such as simple and precise, productivity, creativity and efficiency, saving time,and stimulates and entertains. However, there is very limited study explore the web-based mind maps in teaching the nursing process. Although web-based mind map may has many advantages in improving students’ ability of knowledge memory and information extraction, while there is lack evidence in improving students’ communication skills. Maybe this is because communication skills include a mix of verbal and non-verbal abilities(such as non-verbal cues, body language, facial expressions and physical movement).

Standardized patient (SP) is an individual who is trained to portray a real patient in order to simulate a set of symptoms or problems used for healthcare education, evaluation, and research” [23]. Simulation training with SP was widely used in nursing education, and a number of studies have found that the positive effect in developing interpersonal and communication skills of students [24–26]. Considering the potential advantages of SP, we proposed the mind mapping based on SP so as to make up the shortcomings of simple application of mind mapping.

Therefore, based on web-based mind map, this study integrated SP as a comprehensive method with a view to promote postgraduate nursing students learning in patient education knowledge, communication competence,and self-efficacy in clinical setting.

## Method

### Design

A quasi-experimental design with pretest and posttest model.

### Participants

The inclusion criteria were as follows: (1) aged 18 years or above; (2) All postgraduate nursing students who had taken clinical practicum courses at affiliated hospital of Zunyi Medical University in 2022, (3) willingness to sign an informed consent form. The exclusion criteria included currently participating in another research program.

### Ethical considerations

Ethical approval was granted by the institutional review board of the Affiliated Hospital of Zunyi Medical University. All students were informed about the purpose and procedure of the study and their freedom to withdraw from the study any time was assured. Written informed consent was obtained from all students.

### Process

#### Student recruitment

The postgraduate nursing students who had taken clinical practice at affiliated hospital of Zunyi Medical University were provided a classroom lecture every moth, which organized by nursing department of the hospital every month. After the lecture a research assistant assessed their eligibility for participation. Upon identifying students fulfilling the inclusion criteria, the researcher informed the purpose of this study and obtained their consent to participate. The students were assured the freedom to withdraw from the study at any stage without consequences.

#### Intervention method

In this study, the program was divided into two phases. In the first phase mainly cultured the students ability of summarizing the knowledge of patient education. Basic knowledge training in mind mapping was facilitated by a nursing educator with a PhD and skilled in the application of mind mapping for nursing students. Students are required to attend an intensive one week of face-to-face classes.The training class was conducted for a total of four sessions of 2 h each. For the first two lecture sessions mainly introduce the basic knowledge, charting method of mind mapping, and mind mapping software(“XMind”). For the third session two demonstrations of XMind in patient educational content design were conducted, one was for chronic obstructive pulmonary (COPD) patients and the another one was for Orthopaedic surgery patients. And the educational content design was based on the time points, as for COPD patients mainly included three time points ( admission, in hospital, and discharge), and for Orthopaedic surgery patients mainly included four time points ( admission, before surgery, after surgery, and discharge). And for the last session, each student was asked to choose a disease to complete a design for patient educational content based on mind mapping through XMind software for 40 min, then 6 volunteers of the students were asked to demonstrate their designs and some comments and suggestions from the nursing educator was provided.

In the second phase mainly cultured the students ability of communication in patient education. One week of simulation-based learning was provided for students. Based on the first phase, each student needed to practice two scenarios, which include health education for COPD patient and Orthopaedic surgery patient. The process was facilitated by 6 nursing educators who had experience in simulation-based learning. The students were divided 6 groups, and each group had 11 students who individually practiced with a SP, who was played by the nursing educator. Each student had about 20–25 min for finishing a patient-education session. After that, the nursing educators helped the student analyze their advantage and disadvantages of the communication in patient education, and provided individualized advice to help them handle similar situations in the future.

### Pretest and posttest assessment

The pre and post datas of students’ patient education knowledge and communication competence were collected from clinical educators-assessment, students’ self-efficacy was collected from students self—assessment, patients’ satisfaction was collected from patients-assessment. Pre to the intervention, each student independently provided education for a patient in her or his clinical practice department, and a clinical educator observed the process, then the students, clinical educators, and patients were invited to fill in the assessment. Post to the intervention, the data were collected through the same method.

### Outcome measures

#### Students’ patient education knowledge

A self-designed evaluation questionnaires were developed by the authors in the study to evaluate the.

students’ patient education knowledge. The questionnaires consisted of 6 items, and the score range of each assessment item was 1–6(1: strongly disagree, 2: disagree, 3: mildly disagree, 4: mildly agree, 5: agree, 6: strongly agree). The minimum and maximum score were 6 and 36, respectively. A higher score indicates better patient education knowledge.The questionnaire was tested for reliability and validity prior to the study. The results of the reliability test showed that the Cronbach’s alpha coefficient of the scale was 0.799. The validity of the questionnaire was examined, and the results of KMO and Bartlett’s test of sphericity showed that the KMO was 0.879 and the Bartlett’s test value was 221. 325, which reached the level of significance (*p* < 0.05). It is generally believed that a KMO value greater than 0.6 and a statistically significant difference (*P* < 0.05) can indicate that the questionnaire has a good validity. It is suggested that this questionnaire has good reliability and validity.

#### Students’ patient education communication competence

Students’ patient education communication skills was measured by the Nurse-Patient Communication Competency Rating Scale [27]. The scale was evaluated by the clinical instructor. The scale consists of 42 items and classified into 6 dimensions: to prepare for the interview(6 items), to initiate the communication(7 items), to gather information( 11 items), to share information( 6 items), to elicit and understand the patient’s perspective(6 items ) and to close the interview( 6 items). The scale is a 5-point Likert-type scale in which teachers judge the level of endorsement of items through their own observations, with 1 being “definitely not compliant” and 5 being “definitely compliant.” The total score of the scale ranged from 42 to 210, with higher scores indicating better health education skills. The Cronbach alpha value was 0.953.

#### Students’ self-efficacy

Self-efficacy was evaluated by the General Self-efficacy Scale [28]. The scale consists of 10 items and classified into 4 dimensions:strategic, contingency, motivational, and executive effectiveness. The minimum and maximum score were 10 and 40, respectively. A higher score indicates increased self-efficacy. The total Cronbach alpha values were 0.87.

#### Patients’ satisfaction

Patients’ satisfaction for students’ health education was evaluated using the following question: “Overall, are you satisfied with the students’ health education?” Patients responded as satisfied, neutral, and dissatisfied.

#### Data analysis

The data was analyzed using descriptive and inferential statistics via SPSS 19.0 software. Descriptive and inferential statistics was used to analyze data. *P*-value < 0.05 was denoted statistically significant. Categorical data was described numerically using frequency (percentage) and continuous data using mean (stan- dard deviation, SD). Differences between respondent characteristics pre- and post-intervention were investigated using a paired t-test.

## Results

A total of 74 postgraduate students were enrolled in the trial, and all of them completed the study. Most of the students were female (89.2%), the mean age was 26.72 4.16 years (22–33years) (Table 1).

**Table 1:** Characteristics of postgraduate nursing students (*N*=74)

Variable	Categories	M(SD)	Frequency(N)	Percentage(%)
Gender	Female		66	89.2
	Male		8	10.8
Age		26.72(4.16)		
Ethnic	Han-nationality		63	85.1
	Ethnic minority		11	14.9
Whether the one-child family	Yes		13	17.6
	No		61	82.4

*Note*.M = mean, *SD *Standard deviation

The results of the assessment of postgraduate students’ patient education knowledge before and after intervention are shown in Table 2. The total score for patient education knowledge among nursing students increased from 20.31 ± 2.43 at the baseline to 32.98 ± 2.32 after the intervention. Overall, a statisti- cally significant difference between pre and post-intervention was found for the patient education knowledge (t = 19.473, *p* = 0.000), with the mean dimension score higher post-intervention, indicating improved patient education knowledge overall.

**Table 2:** Comparison of postgraduate nursing students’ patient education knowledge pre- and post-intervention (*N* = 74)

Dimension	Pre-intervention	Post-intervention	T value	*p* value
	M(SD)	M(SD)		
Total score	20.31(2.43)	32.98(2.32)	19.473	0.000^**^
knowledge is accurate	3.18(0.21)	5.38(0.52)	2.177	0.000^**^
knowledge is scientific	3.27(0.33)	5.51(0.31)	−3.241	0.000^**^
knowledge is informative	3.48(0.26)	5.08(0.47)	2.772	0.000^**^
knowledge is instructive	3.52(0.53)	5.63(0.29)	−1.291	0.000^**^
knowledge is suitable for patients	3.77(0.48)	5.72(0.41)	2.007	0.000^**^
knowledge is easy for patients to understand	3.09(0.62)	5.66(0.32)	2.372	0.000^**^

*Note*.M = mean, *SD *Standard deviation

^**^ *p*<0.001

The results of the assessment of postgraduate students’ patient communication competence before and after intervention are shown in Table 3. The total score for patient communication competence among nursing students increased from 163.36 ± 12.78 at the baseline to 197.49 ± 10.67 after the intervention. Overall, a statistically significant difference between pre and post-intervention was found for the patient communication competence (t = 26.432, *p* = 0.000), with the mean dimension score higher post-intervention, indicating improved patient communication competence overall.

**Table 3:** Comparison of postgraduate nursing students’ patient education communication competencepre- and post-intervention (*N* = 74)

Items	Pre-intervention	Post-intervention	T value	*p* value
	M(SD)	M(SD)		
Total score	163.36(12.78)	197.49(10.67)	26.432	0.000**
Prepare for the interview	24.33(2.19)	28.72(1.48)	5.629	0.000**
Initiate the communication	28.73(1.92)	33.17(1.32)	6.381	0.000**
Gather information	42.83(2.58)	51.18(2.21)	12.182	0.000**
Share information	21.35(1.85)	28.42(2.24)	7.282	0.000**
Elicit and understand the patient’s perspective	23.37(1.88)	28.29(1.27)	5.724	0.000**
Close the interview	22.75(2.36)	27.71(2.15)	4.382	0.000**

*Note*. M = mean, *SD *Standard deviation

** *p* < 0.001

The results of the assessment of postgraduate students’ self-efficacy before and after intervention are shown in Table 4. The total score for self-efficacy among nursing students increased from 21.59 ± 3.14 at the baseline to 32.04 ± 4.91 after the intervention. Overall, a statistically significant difference between pre and post-intervention was found for the patient communication competence (t = 12.435, *p* = 0.000), with the mean dimension score higher post-intervention, indicating improved postgraduate students’ self-efficacy overall.

**Table 4:** Comparison of postgraduate nursing students’ self-efficacyin patient education pre- and post-intervention (*N* = 74)

Items	Pre-intervention	Post-intervention	T value	*p* value
	M(SD)	M(SD)		
Total score	21.59(3.14)	32.04(4.91)	12.435	0.000**
Strategic	5.37(0.72)	8.32 (1.26)	5.762	0.003**
Contingency	5.81(0.93)	7.64(0.83)	4.986	0.007**
Motivational	4.78(0.85)	7.56(1.18)	−3.732	0.004**
Executive effectiveness	5.63(0.64)	8.52(1.64)	−6.923	0.004**

*Note*. M = mean, *SD* Standard deviation

** *p* < 0.001

**Fig. 1:**
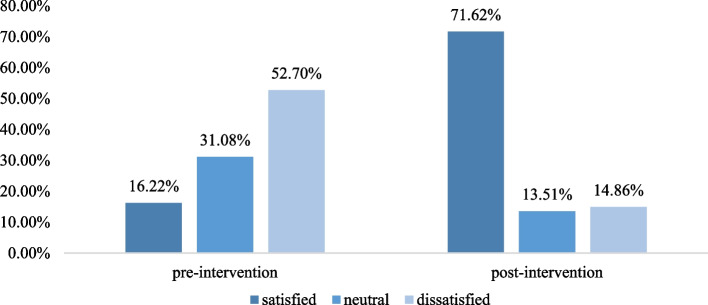
The distribution of patients among the satisfaction patient education pre- and post-prevention

A total of 148 patients were participated to assess the satisfaction for the students’ health education, and 74 for before and after intervention respectively. Figure 1 details the distribution of patients among the satisfaction for students’ health education before and after intervention. Only 16.22%(12/74) patients satisfied with the health education before intervention, and the percentage increased to 71.62%(53/74) after intervention.

## Discussion

To the best of our knowledge, there is very limited study focus on how to improve the patients’ education competence of nursing students in clinical setting. The present study revealed that in the two-weeks mind mapping based on standardized patient intervention significantly improved postgraduate nursing students’ patient education competence during clinical practice. This can be explained by the mechanism underlying each of the intervention’s components.

### Effects on students patient education knowledge

The present study showed that the effects of web-based mind map based on SP program improving students patient education knowledge ability significantly. It has been reported that most nursing students lack of knowledge as one of the main barriers in patient education [11]. While it is crucial to provide a comprehensive, accurate, and clear education knowledge to patients. Extensive clinical experience make the health educators are familiar with the disease management, they could provide perfect education content to patients. However, most of postgraduate nursing students have limited clinical experience, while they provide heath education to patients, they often depend on the memory of theory knowledge. The complex knowledge and information of the diseases make it is a big challenge for students to memory. In current study, we trained students to design web-based mind map for patients’ education knowledge focused on the key time points, as for medical patients included three time points ( admission, in hospital, and discharge), for surgical patients included four time points ( admission, before surgery, after surgery, and discharge). Therefore the students divided the complex education knowledge and information into different parts, and provided the specific education contents at different time point. And this make the knowledge and information more easy to memory. Some studies also reported that mind mapping helped to enhance nursing students’ memory, to better acquire knowledge and analytical abilities and to systematically organize information [17, 18].

### Effects on students patient education communication competence

The results of this study showed that the students’ communication competence in patient education was improved significantly after intervention. An integrative literature review reports that students can experience improved learning outcomes in clinical practice when using simulation with SPs as preparation for practice [24]. Especially the advantage in helping nursing students to develop their communication skills [29]. So present intervention provided the students the opportunity to practice communication skills with the use of SP session, which make the students were familiarized with the communication process. Patients’ feeling is one of the key factors for the effective communication. However, in clinical situation the real patients are unwilling to provide detailed feedback on the process of communication, which may be due to time, illness, privacy, etc. In present study the SPs were played by the nursing educators, so they could not only provide the detailed feeling of the patients during the communication process, and also as education experts they give the suggestion for the advantages and disadvantages in the communication process. Moreover, people’s communication ability is also related to his/her knowledge and information reserve. In present study the web-based mind map not only helps students to memory large number of knowledge and information in short time, but also organization the knowledge and information in a better way. So while nursing students.

communicating with patients they could express the contents of health education comprehensively and systematically.

### Effects on students’ self-efficacy in patient education

This study investigated the students’ self-efficacy in patient education, and the results showed that the mind mapping based on SP program could improve the postgraduate nursing students’ self-efficacy significantly. In present study the students’ self-efficacy in patient education was low at the baseline, the similar report was also found in an early study [30]. In clinical setting, most students feel stress and anxiety while providing health education to patients, they worried that they would not be adequately prepared that they would be unable to explain the I nformation clearly to the patients, or that the patient would simply not want to listen to them [12]. In our previous study found that nursing students’ stress and anxiety were highly correlated with self-efficacy during clinical practice [31]. SP as a way of exposing students to real-life clinical scenarios facilitated students to gain knowledge in a safe environment, reducing the stress and anxiety. Number of studies demonstrated that nursing students decrease anxiety during interaction with patients after interaction with the SPs [32–34]. In present study the students practiced patient education with SPs, this made them familiarize with the process of health education, and also facilitated the communication skill with the patients. Therefore, while the students providing health education to patients in clinical setting the adequate prepare make them feel less stress and anxiety, and improve the self-efficacy.

### Limitations

It must be acknowledged that this study has several limitations. First, this was a pre-post design study, it is possible that not all of the observed changes are due to the interventions–some changes may have been caused by other factors. Second, this study has a single-center design and includes postgraduate nursing students in the affiliated hospital of Zunyi Medical University and, therefore, the results cannot be generalized to the overall population. Third, by using only a quantitative research design, it was not possible to explore students’ perceptions towards the effects of mind mapping based on standardized patient program in the patient education. Fourth, in present study nursing educators were SP could be a bias.

### Implications for nursing education

Lack knowledge and communication competence are the main challenge for nursing students in patient education. Our study highlights the need for training postgraduate nursing students’ knowledge and communication competence in patient education – delivered in mind mapping based on standardized patient program, as each of the method has it’s advantage in improving students knowledge and communication competence. Therefore, the combination of multiple forms of intervention methods is a trend for future interventions.

## Conclusion

The mind mapping based on standardized patient program is an effective way to improve postgraduate nursing students’ knowledge and communication competence in patient education during clinical practice. This is the very limited study to explore the intervention to improve the nursing students’ patient education ability, and the results are encouraging, and can not only help teachers and students understand the potential benefits of combining mind mapping with standardized patient, but also provide a certain reference for educational decision-making and support the use of mind mapping based on standardized patient program in postgraduate nursing education.

## Data Availability

All data generated or analysed during this study are included in this published article.
